# Gaps in the Lexicon Restrict Communication

**DOI:** 10.1162/opmi_a_00089

**Published:** 2023-07-21

**Authors:** Lilia Rissman, Qiawen Liu, Gary Lupyan

**Affiliations:** Department of Psychology, University of Wisconsin–Madison, Madison, WI, USA

**Keywords:** communication, semantics, categories, lexicon, compositionality, Chinese

## Abstract

Across languages, words carve up the world of experience in different ways. For example, English lacks an equivalent to the Chinese superordinate noun *tiáowèipǐn,* which is loosely translated as “ingredients used to season food while cooking.” Do such differences matter? A conventional label may offer a uniquely effective way of communicating. On the other hand, lexical gaps may be easily bridged by the compositional power of language. After all, most of the ideas we want to express do not map onto simple lexical forms. We conducted a referential Director/Matcher communication task with adult speakers of Chinese and English. Directors provided a clue that Matchers used to select words from a word grid. The three target words corresponded to a superordinate term (e.g., *beverages*) in either Chinese or English but not both. We found that Matchers were more accurate at choosing the target words when their language lexicalized the target category. This advantage was driven entirely by the Directors’ use/non-use of the intended superordinate term. The presence of a conventional superordinate had no measurable effect on speakers’ within- or between-category similarity ratings. These results show that the ability to rely on a conventional term is surprisingly important despite the flexibility languages offer to communicate about non-lexicalized categories.

## INTRODUCTION

Two hallmarks of language are conventionality—a shared lexicon of form-meaning mappings, and compositionality—the ability to combine lexical units to create more complex expressions. Speakers can choose more or less compositional strategies. Suppose you are at a restaurant and want something to eat right away. You could suggest to your dining companion that you order “appetizers,” or you could suggest ordering “something small to eat before the main course.” The first strategy relies on the lexical convention *appetizer* (a category is “lexicalized” if it is expressed through a single, non-compositional lexical unit). The second strategy is a more spontaneously generated expression of your wish (sometimes called an ad-hoc description; Barsalou, 1983). But sometimes the first strategy is not available because the conventional term simply does not exist. For example, the English word *seafood* includes both fish and shellfish. The closest Spanish equivalent, *mariscos*, is more accurately glossed as “shellfish” and excludes fish. A Spanish speaker could easily approximate the English meaning by saying *mariscos o pescado* (“shellfish or fish”). Or could they?

We investigated how such differences in lexical conventions affect communication. The lexicon does a lot of work for the people who share it—words are the backbone of communication, they influence memory, and they facilitate category learning (see Kemmerer, 2019; Lupyan, 2012; Lupyan & Zettersten, 2021; Wolff & Holmes, 2011). But how necessary is it to have a conventionalized term for the precise idea one wants to communicate? After all, only a tiny fraction of the categories that people think and communicate about are lexicalized in any given language and it is compositionality that gives language its full expressive power. Do lexical gaps really restrict people’s communication?

Our focus is on superordinate nouns such as *appetizers*, *beverages*, and *crimes*—words that denote categories of basic level nouns and that are often possible to convey through paraphrases and ad-hoc descriptions. We use a cross-linguistic approach, comparing how Chinese- and English-speaking adults communicate about superordinate categories which are lexicalized in one language, but not the other. We test two mechanisms by which the presence of a conventional superordinate term could enhance communication. First, the term could boost communication because language users may be more likely to align in their understanding of the term than in their understanding of a longer phrasal description. Second, the term could boost communication because sharing the term may result in language users representing the members of that category as more similar to each other than they would be otherwise. We test these mechanisms using a Director-Matcher task in which Directors view list of words and provide verbal clues to allow Matchers to select the same words from a larger set (see De Ruiter, 2013 for review).

### Words and Phrases: Different Paths to the Same Meaning?

Toward motivating the hypothesis that people are more aligned in their understanding of a conventional term than a phrasal expression, we review two lines of inquiry: research on whether languages are all equally able to convey the same set of ideas, and research on how linguistic structure is shaped by communicative pressures. Considering the first, vocabularies of different languages carve up the world of human experience in different ways (see Enfield, 2014; Evans & Levinson, 2009; Kemmerer, 2019; Malt & Majid, 2013 for review). For example, Persian lexicalizes in a single word *ænduh* emotions that English speakers distinguish as *grief* and *regret* (Jackson et al., 2019); Spanish speakers use the single label *frasco* for both a squeezable mustard *bottle* and a peanut butter *jar* (Malt et al., 1999, 2003). How do such differences in lexicalization affect communication? Are some ideas more easily expressed in some languages than in others?

Readers may have the intuition that some words are difficult to translate from one language into another (see Eco, 2000; Thompson et al., 2020 for discussion), a sentiment evinced by the Italian expression *traduttore, traditore* (an expression that is itself only loosely captured by its English gloss “the translator is a traitor”). Nonetheless, the view that no language is more or less expressive than any other has a long theoretical tradition in linguistics and cognitive science (see House, 2016 for review). As expressed by Jakobson (1959), “all cognitive experience and its classification is conveyable in any existing language … Languages differ essentially in what they *must* convey and not in what they *may* convey” (p. 234). The engine of this expressive capacity is compositionality—composing words and morphemes into larger phrases, language users can convey an infinite number of thoughts. As articulated by Langacker (1967), “we are perfectly competent of forming and mentally manipulating concepts for which no word is available … For example, imagine a unicorn with a flower growing out of each nostril” (p. 40). Harnad (1996) in turn argues that languages are mutually intertranslatable: “whether it does so analytically, synthetically, or even entirely holophrastically, a language must provide the resources for marking distinctly all the categories we distinguish” (p. 30). More recently, Levinson and Majid (2014) ask whether it makes “a difference to communication accuracy if a notion is lexically codable or not? Perhaps we can do an equally good job at expressing ideas through indirect conveyance” (p. 422). To be clear, this debate assumes that users of different languages have comparable familiarity with the ideas being expressed—a claim of mutual intertranslatability is not defeated by observing that not all cultures readily express notions like “speeding ticket” and “National League pennant.”

Much of what people want to convey cannot be expressed through single words. To convey these non-lexicalized ideas (e.g., ingredients used while cooking to season food) we rely on compositionality. Absent a conventionalized superordinate term, languages provide a range of morphosyntactic strategies that aim to accomplish the same thing as a conventional term (Mauri, 2017; Mauri & Sansò, 2018). For example, Kannada speakers can use a reduplicative strategy: if *pustaka* (‘book’) has a reduplicative marker—*pustaka*-*gistaka*—it conveys ‘books and related stuff’ (Mauri & Sansò, 2018, ex. 15). The theoretical view that languages have equivalent expressive capacity is also supported by people’s general ability to adapt their language to the needs of their situation. For example, hearing people can successfully communicate when forced to use only their hands (Goldin-Meadow et al., 1996; Motamedi et al., 2019) and people modulate their linguistic choices taking into account the needs and backgrounds of their addressees (Clark & Murphy, 1982; Horton & Gerrig, 2002; Suffill et al., 2021). Although morphosyntactic devices vary widely across languages, every language has a rich toolkit for creating compositional meanings and language users can adjust these tools as needed to their situation.

Relatively few studies have empirically measured the power of this toolkit. Experiments on color language show that focal colors are easier to communicate than non-focal colors (Agrillo & Roberson, 2009; Lantz & Stefflre, 1964). For example, Agrillo and Roberson (2009) asked English speakers to communicate a target color to a second group of matchers who had to pick out the target color from a larger color array using only the description. Descriptions were shorter and matchers were more accurate for focal than non-focal colors. As descriptions largely featured basic color terms in combination with modifiers and secondary terms, the results demonstrate that English color language is not equally successful at expressing all regions of color space. In addition, the nameability of colors across languages is weakly correlated across languages, suggesting that languages use different linguistic resources for describing the same regions of color space (Lupyan et al., 2020).

This evidence could be taken to refute the idea that all cognitive experience is conveyable in any language. These studies do not, however, directly address how different languages communicate the same ideas. For regions of conceptual/perceptual space that are successfully communicated in one language (e.g., focal colors), it may be that they are successfully communicated in other languages, just through more syntactically complex expressions. In addition, previous studies have not addressed whether different linguistic strategies make a difference to communication success.

The question of how the presence or absence of a word in one’s lexicon influences communication is also relevant to research on how communicative pressures influence linguistic structure. On the one hand, all languages involve communities jointly creating new linguistic conventions with other people. In Director-Matcher tasks, people initially refer to geometric shapes through spontaneous expressions such as an “upside down martini glass in a wire stand,” but over the course of multiple rounds of communication, converge on much more compact labels like “martini” (Clark & Wilkes-Gibbs, 1986; Hawkins et al., 2020; Krauss & Weinheimer, 1964). Such studies require participants to create new conventions for not-yet-lexicalized ideas. All languages also involve combinatorial rules, which users create by breaking down holistic forms into smaller units which can be generatively recombined (Kirby et al., 2015; Motamedi et al., 2019; Nölle et al., 2018; Raviv et al., 2019). The number of conventional terms that language users share has been argued to be shaped by the need to balance informativeness and complexity, i.e. the lexicon should be large enough to be useful but not so large that it becomes difficult to learn (Gibson et al., 2019; Kemp & Regier, 2012; Zaslavsky et al., 2018). Previous research has not, however, focused on understanding how this dynamic plays out for users of an established linguistic system, who have both conventional terms and compositional rules at their disposal. The Director-Matcher task reported here addresses whether language users communicate more effectively when using conventions vs. more compositional strategies.

### Do Conventional Terms Affect Conceptual Representations?

In addition to asking how conventional terms affect communication through their presence in the linguistic signal, we also ask whether conventional terms shape how language users represent the concepts denoted by the terms. Imagine, for example, a group of people who eat similar foods as in the United States but who use a language, Minglish, that lacks the word *appetizers*. Speakers of American English might be more effective than Minglish speakers at communicating this category because they can use the conventional term *appetizers* rather than longer, non-conventional phrases like *something small to eat before the main course*. It might also be, however, that speakers of American English are more effective at communicating because they represent category members, such as mozzarella sticks, french fries, spring rolls, etc. as more similar to each other than speakers of Minglish do. That is, the referent space being communicated about might differ across languages, facilitating communication even if the conventional term is not part of the signal.

This mechanism is plausible because lexical items have been shown to influence how participants represent the degree of similarity between two objects (Lucy & Gaskins, 2001, 2003). For example, when searching for target objects amid distractor objects in an array, Chinese speakers made more errors when the target and distractor objects received the same classifier morpheme than English and Russian speakers did (where the targets and distractors shared no morphological marking in English and Russian) (Srinivasan, 2010). In an experiment testing for EEG signatures of detection of deviant stimuli, Boutonnet et al. (2013) found that English speakers were more likely than Spanish speakers to perceive infrequent images of cups and mugs as deviant, the explanation being that these images are described through distinct English words (*cup* and *mug*) but only a single Spanish word (*taza*). We are not aware of previous studies testing whether superordinate nouns have such effects on perception of item similarity, or whether such effects are present during a communicative task. Nonetheless, restructuring of semantic space might be one mechanism through which the lexicon facilitates communication.

### Superordinate Nouns Across Languages

Superordinates are a valuable domain for the study of translatability across languages, as people are particularly adept at constructing and interpreting novel category descriptions such as “things to bring with you for a day at the beach.” For example, although English does not lexicalize the category comprising of necklaces, skirts, rings, and hats, the compositional description “things people wear on their bodies” may be sufficient for accurate communication.^1^

Superordinates in one language often lack translational equivalents in other languages (Goddard & Wierzbicka, 2014; Kemmerer, 2019; Mihatsch, 2007; Rosch et al., 1976). For example, the Chinese superordinate *tiáowèipǐn* is loosely translated as “ingredients used to season food” and includes items such as herbs, spices, vinegar, salt, and sugar. Despite the cultural relevance of this category to English-speaking Americans, the Chinese superordinate does not correspond to any English superordinate: *ingredients* is overly broad, containing items such as butter and eggs, while *seasonings* and *flavorings* seem too narrow. Even natural kind categories that may seem cross-linguistically universal are lexicalized in varying ways across languages. For example, Gana divides living things not into categories of *plant* and *animal* but into categories such as *kx’ooxo* (‘living things which are edible’) and *paaxo* (‘living things which are harmful to humans’) (Harrison, 2007).

### Current Study

We used a Director-Matcher paradigm, where one set of participants (the Directors) communicates a set of referents to a second set of participants (the Matchers) (see Kumar et al., 2021). Previous research on color naming shows that not all regions of perceptual space are equally expressible for English speakers. In testing the consequences for communication of having vs. not having a word, a potential pitfall is asking speakers to communicate ideas which are not lexicalized in *any* language and may therefore be linguistically ineffable (Levinson & Majid, 2014). We avoid this pitfall by asking whether Chinese and English speakers communicate more effectively about categories that are lexicalized in their language but are not in the alternate language. We test the somewhat narrower claim that categories which are lexicalized in one language can be conveyed with comparable accuracy by speakers of a different language, who will need to rely on compositional linguistic strategies.

We compared speakers of American English and Chinese for two reasons: first, these languages vary substantially in how their morphemes lexicalize semantic space (Packard, 2000; Saji et al., 2011), and we were able to identify, for each language, superordinates that lacked commonly-used translation equivalents in the other language. Second, there is sufficient cultural overlap between the United States and China that we were able to identify categories that are familiar and culturally relevant for both groups of speakers, even if they are lexicalized in only one of the languages.

## METHODS

### Participants

We recruited 157 participants to play Directors (77 American English speakers: *N*_female_ = 34, *N*_male_ = 42; age *M* = 37; range = 19–63, and 80 Chinese speakers: *N*_female_ = 62, *N*_male_ = 17, age *M* = 22, range = 18–43. An additional 10 English speakers and seven Chinese speakers were tested and excluded for producing clues that referenced targets individually, e.g., “home to gators, sink, small mountains” for the targets *swamp*, *basin*, *hills*. We excluded such clues because the purpose of the experiment was to test how people communicate categories (i.e., regions of conceptual space), rather than a disjoint list of words. Two-hundred ten participants played Matchers: 86 American English speakers: *N*_female_ = 39, *N*_male_ = 47; age *M* = 40; range = 21–70, and 124 Chinese speakers: *N*_female_ = 109, *N*_male_ = 14; age *M* = 23; range = 18–50. An additional English speaker was tested and excluded for failing more than one attention check trial out of five. English speakers were recruited through Amazon Mechanical Turk and were located in the United States. Chinese speakers were recruited through Chinese social media platforms and were located in China. Directors received $2.00/20 RMB and Matchers received $1.50/20 RMB for participating.^2^ Informed consent was obtained for all participants.

### Design and Materials

We identified 10 English and 10 Chinese superordinate terms which we judged to lack a commonly-used translation equivalent in the other language (Table 1). The category denoted by the superordinate term in one language cross-cuts the categories denoted by the labels that are most semantically similar to that term in the other language. For example, the English superordinate *beverage* includes alcoholic drinks such as beer and wine as well as non-alcoholic drinks such as soda. Chinese has distinct terms for alcoholic and non-alcoholic drinks (*jiǔ* vs. *yǐnliào*) and lacks a term that is translationally equivalent to *beverage*.

**Table 1.** English and Chinese superordinate terms tested in Experiment 1.

**English terms (Chinese close translations)**
appetizers (kāiwèicài, light dishes to stimulate the appetite)
beverages (jiǔ, alcoholic drink; yǐnliào, nonalcoholic drink)
crafts (shǒugōng, handicrafts)
crimes (zuìxíng, felony)
drugs (yàopǐn, pharmaceutical drugs; dúpǐn, psychedelic drugs)
pests (hàichóng, damaging insects)
precipitation (jiàngshuǐ, falling water in liquid form)
skills (jìnéng, professional skills; jìqiǎo, tips and tricks)
snacks (xiǎochī, regional cuisine; língshí, processed snacks)
vehicles (chēliàng, motor vehicles; chuán, boat)

**Chinese terms (English gloss)**
nóngchǎnpǐn (agricultural products and livestock)
huàzhuāngpǐn (cosmetics and facial products)
dìxíng (terrain and water features)
jiājù (furniture and home décor)
fúshì (apparel, shoes, and jewelry)
shuǐyù (bodies of water)
shēngwù (living things)
diànqì (electrical appliances and devices)
tiáowèipǐn (food seasonings)
fēngjǐng (scenic places to visit)

*Note*. English terms are accompanied by a list of close translations in Chinese, with glosses of those terms. Chinese terms are accompanied by English glosses.

**Figure 1.**
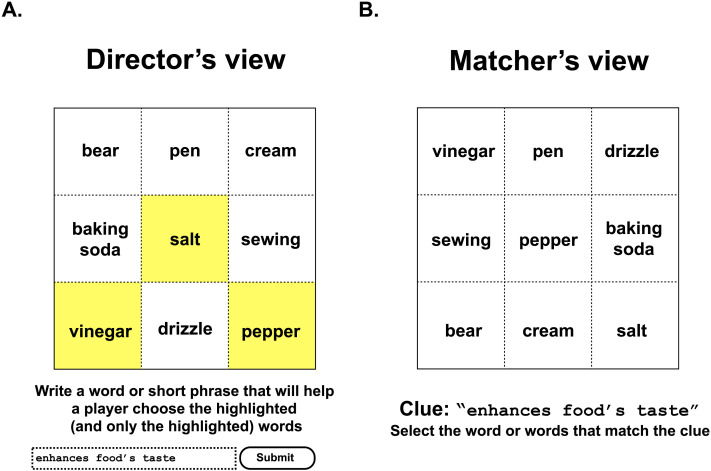
**(A) An example trial shown to a (English-speaking) Director.** (B) An example trial shown to a Matcher yoked to the Director on the left. The targets in this example correspond to the Chinese term *tiáowèipǐn* (‘ingredients used to flavor food’).

To test whether the availability of a superordinate term influences how people communicate about these categories, we asked participants to complete a referential matching task where each participant played either a Director or Matcher role. Directors viewed a 3 × 3 grid with a noun in each cell. Three of these nouns were highlighted and served as targets (Figure 1). The three targets corresponded to a superordinate term in either English or Chinese (e.g., English *beverage* → *wine*, *coffee*, *juice*; Chinese *tiáowèipǐn* ‘ingredients used to flavor food’ → *pepper*, *sugar*, *soy sauce*). The targets for each superordinate cross-cut the labels available in the other language. For example, *beverage* trials did not include target triads such as *soda*, *juice*, *sparkling water*, as this triad could be expressed through the Chinese superordinate term *yǐnliào* (‘non-alcoholic drinks’). The targets were all typical examples of the superordinate. We asked English and Chinese speakers to list six members of each superordinate category in Table 1 and we used these norms to select the targets for each superordinate.

At the request of an anonymous reviewer, we conducted a norming study in which 70 English and 59 Chinese speakers evaluated whether the targets were good examples of the superordinate terms. For example, English speakers rated on a 1–5 point scale whether they agreed with the statement “soda is a type of beverage.” These participants also rated whether the targets were good examples of close, single word translations of the superordinate terms in the alternate language. For example, Chinese speakers rated whether they agreed that soda is a type of jiǔ (‘alcoholic drink’), where *jiǔ* is a close translation of *beverage*. Conversely, English speakers rated whether they agreed that vinegar is a type of condiment, where *condiment* is a close translation of *tiáowèipǐn*. For some of the superordinate terms, we tested multiple close translations (e.g., both *jiǔ* (‘alcoholic drink’) and *yǐnliào* (‘non-alcoholic drink’) for *beverages*). We collected ratings for all target/superordinate and target/close translation pairs. We found that ratings were higher for the superordinate terms than the close translations (English terms: 4.6 vs. 3.7, *β* = .69, 95% CI = [.30, 1.08], *t* = 3.47, *p* < .01; Chinese terms: 4.4 vs. 4.0, *β* = .31, 95% CI = [.07, .54], *t* = 2.55, *p* < .05). At the same time, for one of the English terms and four of the Chinese terms, ratings of the close translations were as high as the superordinate terms (e.g., the ratings for English *apparel* were similar to the ratings for Chinese *fúshì*). We return to differences across individual terms in Which Categories Were Easiest and Hardest to Convey? section. Full norming data and visualizations are available in Table S3 and Figure S2 in Supplementary Materials.

In addition to the targets, the grids included two other types of nouns: two lures, which were semantically similar to the targets, but were not members of the target superordinate category (e.g., for *beverage* these were *vinegar* and *oil*), and four unrelated words, which were semantically dissimilar to the targets and lures (e.g., *motorcycle*, *star*, *tree*, *sleet*). Lures were designed to make it difficult for participants to describe the targets in terms of labels more general than the superordinate—for example, to describe *wine*, *coffee*, *juice* as “liquids” rather than as “beverages.” Unrelated words for each superordinate were selected randomly from the set of targets and lures for other superordinates. Another consideration for selecting the items was that they be familiar to both English and Chinese speakers, e.g., beer but not oregano is common in both the US and China. Lures and unrelated words were selected by the first and second authors according to these criteria.

We constructed six unique grids for each superordinate term. The set of three targets differed across the six grids but individual nouns sometimes appeared in multiple grids. For example, the targets were *wine*, *coffee*, *juice* in one *beverage* grid and *coffee*, *beer*, *tea* in another. The grids for each English and Chinese superordinate were translated into Chinese and English, respectively. Directors and Matchers saw two grids per superordinate and each grid contained different items. We constructed nine combinations of grids such that the particular grids seen for *beverages* and *tiáowèipǐn*, for example, were not always the same.

### Procedure

Directors were instructed to write a clue that would enable another person to choose the highlighted and only the highlighted words from the same grid. An error message appeared if Director’s clue contained any of the words in the grid. Each Director viewed 40 trials, presented in a random order. The position of each word in the 3 × 3 grid was randomized. The Matching task was conducted after the Director task. Matchers were yoked to a particular Director and saw the same grids as that Director (without highlighting and with the items re-ordered). Matchers were asked to select the word or words that match the clue. Matchers completed five attention check trials where they were asked to pick a particular word from the grid (e.g., selecting *purple* from a grid containing color words). All studies were conducted online.

### Director Coding

To understand what kinds of clues were produced (and to subsequently link these to Matchers’ success rates), we coded each clue produced by each Director into several categories (Table 2).

**Table 2.** Coding categories for Directors’ clues with descriptions and English examples.

**Category**	**Description**	**Examples**
Intended Superordinate	The intended superordinate term from Table 1	“vehicles”
		“types of precipitation”
Modified Intended Superordinate	The intended superordinate term is used but is modified	“greasy appetizers”
		“sweet or salty snacks”
		“skills that require learning”
Other Superordinate	A single superordinate term that differs from the intended term	“accessories”
		“drinks”
		“food”
Modified Other Superordinate	A superordinate term other than the intended term is modified	“food ingredients”
		“animals that are annoying”
		“common ingredients of a chinese glaze style sauce”
Set Operational	A category based on the intersection, union, and/or complementation of multiple superordinate terms	“insects and rodents”
		“clothing or hair products”
Property	A property or constitutive feature of the targets	“sneaky”
		“good on chips”
		“water”
Thing-Property	A phrase where the targets are described as things having a particular property	“living things”
		“things that have legs”
		“things you can use for your hobbies”
Context Association	A word or phrase that is associated with the targets but is not a property or superordinate term	“bad fairy tale”
		“nature”
		“Game of thrones”
Additional Exemplar	A word or phrase that is a member of the same category as the targets	“valley” (for targets *swamp*, *hills*, *basin*)

Chinese Directors sometimes produced variations of the intended superordinate—for example, *tiáowèiliào* or *tiáoliào* could be used instead of *tiáowèipǐn*. We coded these as being an instance of the intended superordinate, as these variations can be used interchangeably. As Chinese orthography lacks word boundaries, we applied Packard’s (2000) compositionality criterion to determine if a clue should be coded as superordinate or modified superordinate. That is, if the meaning of a clue is not compositionally transparent from its morphological structure and if it would lose its idiomatic meaning by changing any of its morphemes, it was coded as a superordinate. For example, for a *tiáowèipǐn* trial, the clue *jiàngliào* (‘sauce’) was coded as Other Superordinate but the clue *chúfáng yuáncáiliào* (‘kitchen raw materials’) was coded as Modified Other Superordinate.

Clues were coded by the first and second authors and by one English-speaking and one Chinese-speaking research assistant. Clues which were not obviously instances of any of the categories in Table 2 were discussed as a group and assigned a category.

### Similarity Ratings

To assess whether the presence of a superordinate term was related to how speakers represent the similarity space of the nouns in each category (see Do Conventional Terms Affect Conceptual Representations? section), we asked English and Chinese speakers to rate the pairwise similarity between the nouns comprising the target categories. We recruited 124 participants (62 English speakers: *N*_female_ = 25, *N*_male_ = 36; age *M* = 40; range = 21–67, and 62 Chinese speakers: *N*_female_ = 40, *N*_male_ = 14; age *M* = 25; range = 18–67). An additional three English and 11 Chinese speakers were tested but were excluded for being a non-native speaker (of English: *N* = 1) or for failing one or more attention check trials out of four (English: *N* = 2; Chinese: *N* = 11). English speakers were recruited through Amazon Mechanical Turk and were located in the United States; Chinese speakers were recruited through Chinese social media platforms and were located in China. Participants received $1.50/9 RMB and an additional $1/7 RMB bonus if they rated two sets of word pairs.

For each language, we collected ratings for 854 word pairs (e.g., *beer-soda*) out of a total of 1800 word pairs across all grids. We collected ratings for all target-target pairs (360 pairs per language). We also collected ratings for target-lure pairs (e.g., *beer-vinegar*) in which the lure was chosen on six or more trials of the Matching task by either English or Chinese speakers (334 pairs per language). We additionally collected ratings for 160 target-unrelated pairs in each language (e.g., *beer-sky*). We tested four randomly selected target-unrelated pairs per term—these pairs were included to increase the diversity of the pairs being rated. We omitted collecting ratings for the majority of target-unrelated pairs, as non-targets were almost never chosen by the Matchers. The target-target, target-lure, and target-unrelated pairs were evenly distributed across 18 lists, such that each list contained 80–88 pairs.

English participants were shown the following instructions: “In each trial, you will see two words, where each word refers to a thing. You will rate how similar these two things are to each other. For example, if you saw the words: *cat-dog* or *newspaper-magazine*, you should rate these things as being fairly similar to each other. On the other hand, if you saw the words: *cat-crocodile* or *newspaper-truck*, you should rate these things as being fairly dissimilar to each other.” Chinese participants were given parallel instructions with translational equivalents of the English example words. Each pair was rated on a scale from 1 (not similar at all) to 5 (very similar). To address possible issues of scale bias across participants, we z-transformed each participant’s set of ratings.

## RESULTS

### Analytic Approach

We fit mixed effects linear regression models using the *lme4* package for R (Bates et al., 2014; R Core Team, 2022). We used sum contrast coding for the independent variables Speaker Language (Chinese speakers vs. English speakers) and Term Language (implicit Chinese superordinate terms vs. implicit English superordinate terms). Unless noted otherwise, models included random intercepts for Participants and Implicit Terms as well as Participant by Term Language random slopes and Implicit Term by Speaker Language random slopes. Coefficient values for the Speaker Language and Term Language variables reflect the difference between Chinese speakers/Chinese terms and the overall mean for each variable. Continuous variables were centered and scaled. We used the *lmerTest* package (Kuznetsova et al., 2017) and Satterthwaite approximation to compute *p*-values for fixed effects (see Luke, 2017). Matcher trials with reaction times greater than 25,000 ms were excluded from analysis. 95% confidence intervals were calculated using *summarySEwithin* from the *Rmisc* package. Stimuli materials, data files, analysis scripts, model syntax, and Supplementary Materials are available at: https://osf.io/cdg4j/.

In How Successful Were the Matchers? section, we test whether communication is more accurate when a shared superordinate term is available. In How Did Directors’ Clues Affect Matchers’ Accuracy? to What Alternatives to the Intended Superordinate Did People Use? sections, we test whether the observed boost in communication is due to properties of the linguistic signal itself—whether Directors use this shared term rather than other clues, such as ad-hoc, phrasal descriptions. In Did Word Pair Similarity Ratings Differ Across Languages? section, we ask whether superordinate terms influence participants’ similarity ratings of the words in the grids. This analysis addresses whether the observed communication boost is due to shared terms influencing how language users represent the members of each superordinate category.

### How Successful Were the Matchers?

**Figure 2.**
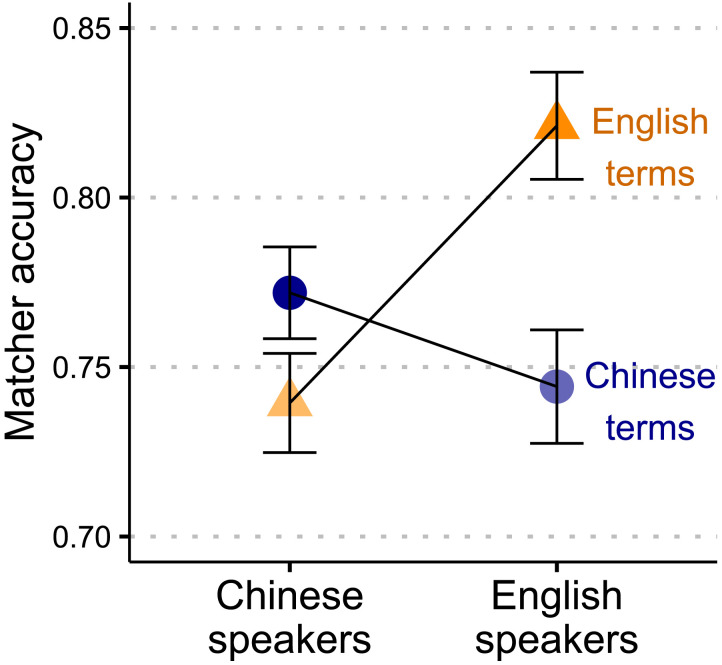
**Mean Matcher accuracy across Speaker Languages and Term Languages.** Error bars denote 95% confidence intervals of the mean.

Matchers’ mean accuracy (calculated as their hit rate minus their total false alarm rate) was 76.7% (95% CI = [76.0%, 77.2%]). Mean accuracy across speaker languages and term languages is shown in Figure 2. Table 3 shows performance broken down by hit rates, lure false alarm rates, and unrelated word false alarm rates across speaker languages and term languages. Hit rates were computed as the number of targets selected divided by 3. Lure false alarm rates were the number of lures selected divided by 2; unrelated word false alarm rates were the number of unrelated words selected divided by 4; total false alarm rates were the number of lures and unrelated words selected divided by 6.

**Table 3.** Mean hit rates, lure false alarm rates, and unrelated word false alarm rates for each language and term type; 95% confidence intervals of the mean are shown in parentheses.

	**Hit rate (targets)**		**False alarm rate (lures)**		**False alarm rate (unrelated words)**	
	**Chinese speakers**	**English speakers**	**Chinese speakers**	**English speakers**	**Chinese speakers**	**English speakers**
Chinese terms	.87 (.01)	.82 (.01)	.22 (.02)	.24 (.02)	.02 (.006)	.03 (.005)
English terms	.83 (.01)	.88 (.01)	.21 (.02)	.19 (.02)	.02 (.005)	.02 (.003)

To test whether Matcher accuracy was reliably different across these groups, we began with a linear mixed effects model that included Speaker Language and Term Language and their interaction as fixed effects. There were no main effects of Speaker Language or Term Language (*p*s > .1), but there was a significant Speaker Language-by-Term Language interaction (*β* = .10, 95% CI = [.05, .15], *t* = 3.9, *p* < .01). This indicates that Matchers were more successful for trials corresponding to a conventional term in their language. We will refer to this finding from now on as the congruency advantage.

We next examined the contribution of the similarity between each of the targets (target-target similarity) and the similarity between the targets and the lures (target-lure similarity). We calculated these estimates using the pairwise similarity ratings described in Similarity Ratings section (e.g., how similar *beer* and *soda* are to each other). The target-target similarity for each grid was the average of the similarity ratings for each pair of targets. Target-lure similarity for each grid was the average of the similarity ratings for each target-lure pair. As we might expect, accuracy was significantly higher for trials with higher target-target similarity (*β* = .57, 95% CI = [.49, .64], *t* = 14.6, *p* < .001), and accuracy was significantly lower for trials with higher target-lure similarity (*β* = −.14, 95% CI = [−0.2, −0.06], *t* = −3.5, *p* < .001). There was no interaction between either similarity type and Speaker- or Term Language (*p*s > .1). Although adding the similarity ratings improved the fit of the accuracy model, the size of the congruency advantage was comparable to a model without the similarity rating predictors (*β* = .09, 95% CI = [.04, .15], *t* = 3.2, *p* < .01). This indicates that the congruency advantage is not mediated by the similarity structure of the items in the grid.

### How Did Directors’ Clues Affect Matchers’ Accuracy?

**Figure 3.**
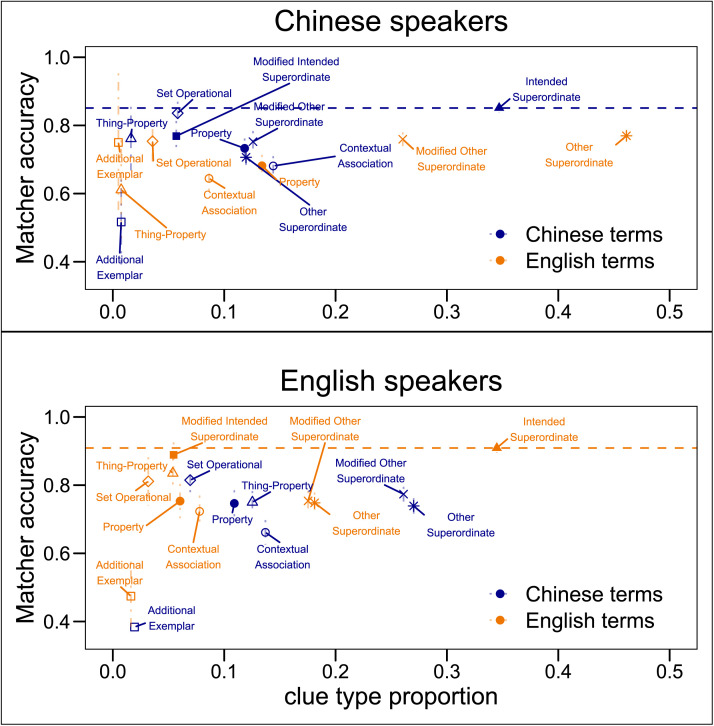
**The relationship between clue type proportion (*x* axis) and Matcher accuracy (*y* axis) for Chinese speakers (top) and English speakers (bottom) broken down by Term Language.** Performance for categories lexicalized in Chinese in blue; categories lexicalized in English in orange. By definition, Intended Superordinate and Modified Intended Superordinate clues were only possible for congruent trials, i.e., blue for Chinese speakers and orange for English speakers. Dashed error bars denote 95% confidence intervals of the mean. Dashed horizontal lines show mean Matcher accuracy for Intended Superordinate clues for each speaker group.

How did Matchers perform given different types of clues? Setting aside for the moment Directors’ use of Intended Superordinates (i.e., using *appetizers* for the category comprising *French fries*, *onion rings*, and *chicken wings*), we find that the three most common types of clues were: Other Superordinates (e.g., *food* for the appetizer category), 26%; Modified Other Superordinate (e.g., *fried foods*), 21%, and Contextual Association (e.g., *olive garden*), 11%. These three types of clues had Matcher accuracies corresponding to, respectively, 75%, 76%, and 67%. Excluding Intended Superordinates and Modified Intended Superordinates, the relationship between clue-type frequency and Matcher accuracy was *r*(26) = .30, *p* > .1 (see Figure 3).

By definition, producing an Intended Superordinate or a Modified Intended Superordinate was only possible in a congruent trial. For these trials, the single most common type of clue was Intended Superordinate, for both Chinese and English speakers (35% and 34% of clues, respectively). The use of Intended Superordinates corresponded to highest Matcher accuracies (see Figure 3). To assess how accuracy for each clue type differed from accuracy for Intended Superordinates, we fit separate linear mixed effects models for Chinese and English speakers. Among English speakers, the use of Intended Superordinate clues led to significantly higher accuracy than all the other clue types (*p*s < .01) except for Modified Intended Superordinate (e.g., *greasy appetizers*) (*p*s > .1). Among Chinese speakers, Intended Superordinate clues also led to significantly higher accuracy than all the other clue types (*p*s < .01) except for Modified Intended Superordinate and Set Operational (e.g., *seafood and sides*) clues (*p*s > .1). See Table S1 in Supplementary Materials for full model specification. These results show that, on average, Directors were most successful at conveying the intended category when they used the intended superordinate term.

It may be that the relatively lower success of the alternate strategies is due in part to participants being less familiar with the categories in the incongruent trials. If, for example, an English Director is viewing a trial in the Chinese term condition and produces an Other Superordinate clue, accuracy could be lower not because of the clue itself, but because English Matchers are less disposed to view the targets as part of a single category (lacking an English term corresponding to this category). To more directly test the communicative efficacy of the intended superordinates, we examined performance on just the congruent trials (i.e., English categories for English speakers; Chinese categories for Chinese speakers). In Chinese, accuracy was significantly higher for Intended Superordinates (*p*s < .01) than for all strategies except Modified Intended Superordinate, Set Operational, and Thing-Property (*p*s > .1). In English, accuracy was higher for Intended Superordinates (*p*s < .01) than for all strategies except Modified Intended Superordinate and Set Operational (*p*s > .1). See Table S2 in Supplementary Materials for full model specification. These results show that even when the target categories were reflected in the speakers’ lexicons, most alternate strategies used by the Directors were not as effective as the intended superordinate in conveying the category.

**Figure 4.**
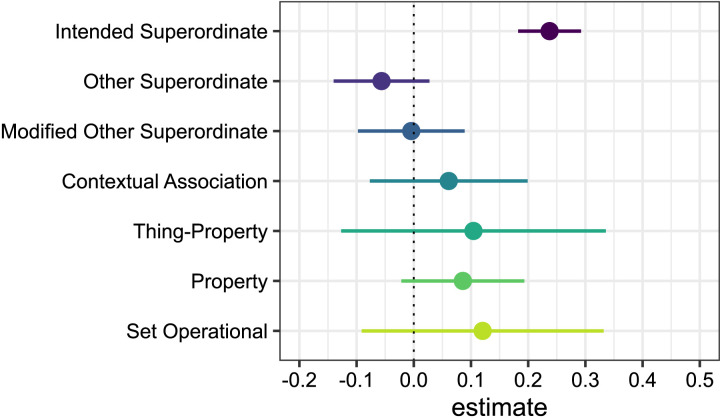
**Congruency advantage by code type.** Model coefficient estimates and 95% confidence intervals for the Speaker Language by Term Language interaction term for each code type. Code types are ordered by overall frequency. The coefficient estimate for “intended superordinate” includes both Intended Superordinates and Modified Intended Superordinates. We did not compute a model for Additional Exemplar clues as these were too infrequent.

Matchers were more accurate when shown grids with items that could be named by a superordinate term in their language. In addition, accuracy was highest when the clue contained the intended superordinate, which was only possible in the congruent conditions. As a direct test of how the congruency advantage was driven by the particular clues that the Directors provided, we examined the size of the interaction term between Speaker Language and Term Language for the different clue types. We compared these interaction terms to an *intended term* baseline computed by comparing accuracy for congruent trials that used an intended superordinate term with all incongruent trials. Figure 4 shows model coefficients and 95% confidence intervals for each clue type.

As described in Do Conventional Terms Affect Conceptual Representations? section, the presence of a label can attune language users to the common structure shared by items that are members of that category. Directors and Matchers could have shared more similar representations of the categories in congruent than incongruent trials, leading to better Matching accuracy regardless of what clue the Director provided. The pattern shown in Figure 4 argues against this mechanism. We observed no congruency advantage for any clue type besides Intended Superordinate/Modified Intended Superordinate. This suggests that the congruency advantage was driven entirely by use of the intended superordinates.

Language users may be able to overcome the absence of a conventional term in their language by using longer, more morphologically complex expressions. As a measure of the complexity of Directors’ clues, we calculated the length of each clue (mean number of characters for Chinese clues; mean number of words for English clues; see Figure S1 in Supplementary Materials). Directors largely confined themselves to single words or short phrases: mean clue length was 3.5 characters in Chinese and 2.2 words in English. In Chinese, the longest types of clues were Modified Other Superordinate and Set Operational (*M* = 5.6 characters each); in English, Set Operational and Thing-Property clues (*M* = 4.3 and 4.2 words, respectively). We tested whether Directors produce longer clues when they are asked to communicate about a category that is not conventionally named in their language. Chinese clues were significantly longer than English clues (which is a trivial finding because length for Chinese clues was measured in characters, not words) (*β* = .28, 95% CI = [.19, .38], *t* = 5.64, *p* < .001); there was no significant effect of Term Language on clue length (*β* = .011, 95% CI = [−.063, .086], *t* = .29, *p* > .1). When tasked with communicating a category conventionalized in their language (trials in which the Intended Superordinate was available), Directors produced significantly shorter clues (*β* = −.073, 95% CI = [−.12, −.027], *t* = −3.11, *p* < .01). Overall, clue length did not predict accuracy, either as a linear predictor (*β* = −.01, 95% CI = [−.037, .017], *t* = −.74, *p* > .1) or a quadratic predictor (*β* = −.000, 95% CI = [−.024, .024], *t* = −.012, *p* > .1). Simply producing longer clues did not make communication more successful in this task.

### Which Categories Were Easiest and Hardest to Convey?

**Figure 5.**
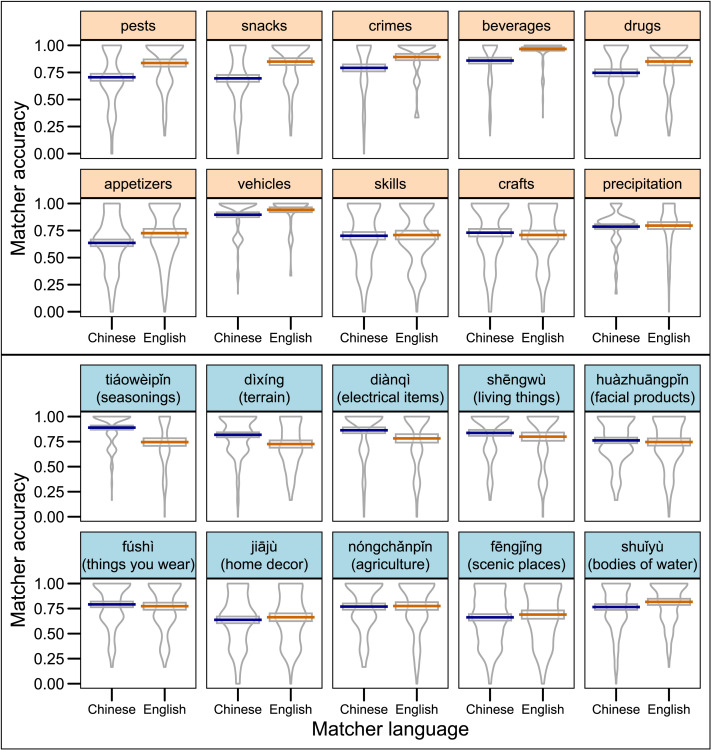
**Matcher accuracy for Chinese vs. English speakers for each superordinate term.** English terms are shown in the upper panel and Chinese terms in the lower panel. Colored lines show the mean for each group and boxes show 95% confidence intervals of the mean. Within each language, individual term panels are ordered by the size of their congruency effect, from largest to smallest.

The categories we used span biological categories, human artifacts, and culturally-specific types of food. Although *Term* was included as a random effect in the above models, examining how accuracy varied for the different terms allows us to see if access to a conventional superordinate was more critical for some terms than others. Figure 5 shows the difference between Chinese and English Matcher accuracy for each term. Descriptively, most English terms showed the congruency advantage. Congruency was more variable for Chinese terms, as Chinese speakers showed a strong congruency advantage for only four of the ten terms.

**Figure 6.**
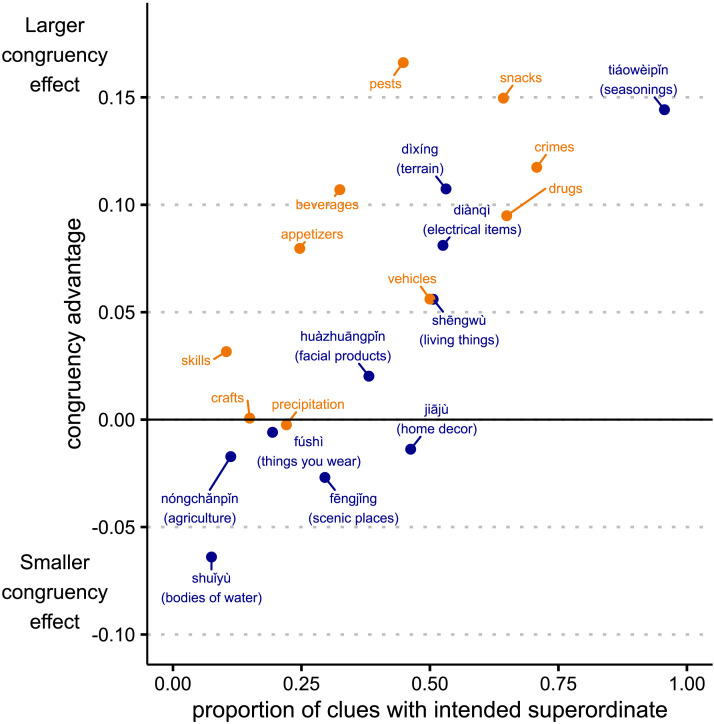
**Proportion of trials where the Director used the intended superordinate (either Intended Superordinate or Modified Intended Superordinate) vs. Matcher congruency advantage for each superordinate term.** The congruency advantage is: for Chinese terms, Chinese Matcher accuracy minus English Matcher accuracy; for English terms, English Matcher accuracy minus Chinese Matcher accuracy.

To better understand the variability that we observed across terms, we analyzed the relationship between the congruency advantage for each term and Directors’ propensity to use the intended superordinate for that term. For this analysis, we counted both Intended Superordinate and Modified Intended Superordinate clues as using the intended superordinate, as these two strategies did not lead to significantly different Matcher accuracy. Across individual terms, use of the intended superordinate ranged widely, from 96% of clues (*tiáowèipǐn* ‘seasonings’) to 8% (*shuǐyù* ‘bodies of water’). Whether the intended superordinate was used for a particular term was strongly positively correlated with the size of the congruency advantage for that term (*r*(18) = .74, 95% CI = [.44, .89], *p* < .001; see Figure 6). This result aligns closely with the analyses in the previous section: the congruency advantage is due to the Directors’ actual use of the intended superordinate. When this term was not used, Chinese and English speakers had comparable rates of success in using alternate strategies to convey the categories.

As described in Design and Materials section, we collected norms of how good each target was as an example of each intended superordinate term (e.g., whether soda is a type of beverage). There was a moderate, marginally significant correlation between these ratings and how often Directors used each intended superordinate: *r*(18) = .39, 95% CI = [−.06, .71], *p* = .08). To assess whether there was relationship between the norms and the congruency advantage by term, we computed the norm advantage for each term—the difference between the ratings for the intended superordinate and the ratings for the best translation (e.g., *beverages* vs. *yǐnliào* ‘non-alcoholic drink’). There was a moderate correlation between the norm advantage and the congruency advantage for each term: *r*(18) = .51, 95% CI = [.09, .78], *p* < .05. We fit a single model of the congruency effect, asking whether the norm advantage and Directors’ propensity to use the intended superordinate made independent contributions. There was a marginally significant effect of norm advantage (*β* = 0.31, 95% CI = [−.024, .64], *t* = 1.95, *p* = .07), suggesting that when lexical gaps are greater (i.e., a set of targets is well lexicalized in one language but not another), Matchers may have more difficulty reconstructing the categories seen by the Directors. However, the variability in congruency effect that we observed across terms was primarily explained by whether the intended superordinate terms were used by Directors (*β* = 0.64, 95% CI = [.30, .97], *t* = 4.05, *p* < .001).

Given the demonstrated advantage of using the intended superordinate, we asked how consistent this strategy was across participants. Directors’ likelihood of using the intended superordinate in congruent trials ranged from 0% to 75% (1st quartile = 30%; 3rd quartile = 50%). We return to this point in the Discussion, when we consider how individuals might differ in their choice of strategies and how this is relevant to theories of communication.

### What Alternatives to the Intended Superordinate Did People Use?

The analyses thus far show that use of the intended superordinate has a robust effect on communication: in both languages, Matchers are more accurate when the Director uses the intended term. At the same time, intended superordinates were used on fewer than half of the congruent trials. So, even when speakers had the intended term available to them (at least in principle) they used a variety of other strategies which sometimes led to perfect accuracy.

As a demonstration of the range of alternate strategies that Directors used, Table 4 shows some of the English clues for *appetizers* trials that led to perfect accuracy. For this example, the targets were *French fries*, *onion rings*, and *chicken wings* and the lures were *hamburger* and *broccoli*. Although *appetizers* was the modal clue for these trials, Directors often used a flexible, non-conventional description, drawing on other superordinates (*snacks*, *sides*, *foods*) in conjunction with different properties that the targets share (being fried, being salty, being unhealthy). Table 4 also shows some of the Chinese clues for *fēngjǐng* (‘scenic places to visit’) that led to 100% accuracy. For these clues, the targets were *lake*, *forest*, and *castle* and the lures were *soil* and *land*. In addition to using other superordinates like *dìfāng* (‘places’) and *jǐngdiǎn* (‘places of interest’), Chinese Directors also provided contextual clues integrating the targets, as with *bīngxuěqíyuán* (‘Frozen’), and highlighted specific features of the targets, such as being ancient and mysterious.

**Table 4.** Example English and Chinese clues that led to 100% Matcher accuracy.

**Example clues for *appetizers***	**Example clues for *fēngjǐng* (scenic places to visit)**
fast food snacks that have no beef	chángjiànde jǐngqū (‘common places of interest’)
deep fried	gǔlǎo ér shénmìde dìfāng (‘ancient and mysterious places’)
salty foods without a bun	jǐngdiǎn (‘places of interest’)
unhealthy sides	Zìrán hé rénwén fēngjǐng (‘natural and cultural scenic places to visit’)
these are common snacks you can share	bīngxuěqíyuán (‘Frozen’; i.e., film name)
cooked in oil	

These examples demonstrate that even when a conventional term was available to communicate a category, Directors sometimes chose to highlight more idiosyncratic dimensions along which the targets were similar to each other, and that these compositional descriptions sometimes resulted in perfect accuracy.

### Did Word Pair Similarity Ratings Differ Across Languages?

In our analyses of Matcher accuracy, we included mean target-target and target-lure similarity ratings as predictors (these ratings are described in Similarity Ratings section). As expected, target-target pairs were rated as more similar (*M* = .36) than target-lure pairs (*M* = −.14) (*β* = .48, 95% CI = [.46, .51], *t* = 32.7, *p* < .001). In a final analysis, we ask whether the similarity ratings are themselves influenced by the presence of a superordinate. That is, do speakers judge that two targets are more similar to each other when they are part of an intended superordinate category than when they are not? To test this question, we analyzed the ratings for individual target-target pairs across Speaker Languages and Term Languages.

English target-target pairs were rated as more similar than Chinese target-target pairs (*M* = .49 vs. *M* = .24), but this difference was not statistically significant (*β* = −.12, 95% CI = [−.29, .045], *t* = −1.44, *p* > .1).^3^ English ratings were not different overall than Chinese ratings (*M* = .36 vs. *M* = .37; *β* = .007, 95% CI = [−.04, .058], *t* = .27, *p* > .1). The crucial question is whether there was a Speaker Language by Term Language interaction such that the same pairs were rated as more similar for congruent trials. This interaction was not statistically significant (*β* = .041, 95% CI = [−.012, .093], *t* = 1.5, *p* > .1), suggesting that the superordinate terms in our study do not affect how Chinese and English raters represent the similarity space of the items in the grids. For ratings of target-lure similarity, there were no significant effects of Speaker Language, Term Language, or their interaction (*p*s > .1).^4^

## GENERAL DISCUSSION

Languages differ in the categories they express using conventional terms. Because languages are compositional, ideas not lexicalized through a conventional term (e.g., “comfort TV shows”) can nevertheless be expressed. Does the generative, compositional capacity of language allow an idea lexicalized in one language to be expressed equally well in languages that do not lexicalize this idea? We investigated the consequences of having vs. not having a conventional term on speakers’ ability to communicate superordinate categories. We found that conventional terms matter. Matchers were more accurate when they had to infer a category that had a conventional label in their language (i.e., there was a congruency advantage). The strength of the congruency advantage across individual terms was strongly correlated with Directors’ propensity to use the intended superordinate. The congruency advantage disappeared when the Director did not use the intended superordinate term.

Cross-linguistic differences have been shown to affect how language users represent the similarity of object tokens in non-linguistic tasks (Do Conventional Terms Affect Conceptual Representations? section). We did not find evidence for such an effect in this task—there was no congruency advantage when the clue did not contain the intended superordinate. In addition, there was no congruency advantage in the pairwise similarity rating task. For example, English speakers rated the similarity between items comprising the category *dìxíng* (e.g., *mountain*/*valley*, *valley*/*river*, etc.) as similarly as Chinese speakers did, despite not having a conventional term. These results suggest that Chinese speakers do not represent *mountain*, *valley*, and *river* as being more conceptually unified than English speakers do, even though this category is lexicalized in Chinese but not English. Given the limitations on what can be inferred from such a null result, further experimentation is needed to gain a fuller understanding of whether superordinate terms can restructure how objects are conceptualized. Further experimentation is also needed to address whether conventional terms would affect similarity ratings if the words were viewed in the context of the entire nine word grid, rather than in isolated pairs (see Goldstone, 1994).

Our results show that despite the flexibility languages afford us, there is a limitation on how well people can communicate in the absence of a conventional label. This limitation is at the practical level rather than the absolute level. It is entirely possible that an experienced translator, with native language experience in both English and Chinese and with suitable cultural knowledge, may be able to compensate for the lack of a conventional superordinate term. It is also possible that in an interactive context that allows for repair, speakers can quickly align to avoid the kinds of miscommunication we observed in our non-interactive tasks. That said, even a small advantage for interpreting conventional terms over ad-hoc phrases may result in a substantial cumulative advantage in real-world situations where repair can be costly and speed is of the essence.

Using the intended superordinate puts the Director/Matcher pair at a decided advantage in our tasks. Despite the clear benefit of using superordinates, Directors in congruent trials used the intended superordinate as a clue less than 50% of the time. Our norming data show that on average, the targets were rated as good examples of each superordinate term (receiving an average rating of at least 4.0 on a scale from 1 to 5; see Figure S2). Non-use of the intended superordinate may therefore be a partial consequence of multiple superordinates being available that share similar meanings (e.g., some English Directors used the clue *drinks* instead of *beverages*). The Other Superordinate strategy was used on only 16% of congruent trials, however. On the remaining 44% of the trials, Directors used longer, compositional descriptions. We speculated at the outset that if language users share a short, conventional term, then in a communicative task such as ours, they will primarily use this term. The actual range of clues that Directors used was more variable than we expected.

Being able to flexibly describe categories through compositional expressions is clearly important because most ideas that we want to convey in everyday life are not lexicalized. This flexibility allowed Chinese and English speakers to be moderately successful in the incongruent conditions, with Matchers achieving 74% accuracy. Less intuitively, flexibility appears to remain important even when one’s language does lexicalize a particular idea. Directors used the intended superordinate on less than half of congruent trials even though avoiding the intended superordinate came at the cost of lower accuracy. This suggests that combinatorial strategies beyond the intended superordinate confer their own type of communicative advantage.

A prominent viewpoint is that communication is structured to maximize efficiency (Gibson et al., 2019; Kemp et al., 2018; Piantadosi et al., 2011; Rubio-Fernandez & Jara-Ettinger, 2020; Trott & Bergen, 2022). As described by Gibson et al. (2019), “Efficiency in communication means that successful communication can be achieved with minimal effort on average by the sender and receiver. Typically, effort is quantified using the length of messages, so efficient communication means that signals are short on average while maximizing the rate of communicative success” (391). While Directors used the most efficient type of signal (the intended superordinate) on a plurality of congruent trials, this framework does not readily explain variability at the level of individual participants and trials. For instance, why would Directors often choose to produce longer, compositional descriptions when single word conventional terms are available, especially given that the conventional terms result in higher accuracy? Directors would of course be expected to use longer over shorter clues if they believed this would lead to a higher probability of success, but it is unclear why the clues in Table 4, such as *cooked in oil*, for example, would be more successful than the conventional term *appetizers*. In addition, individual Directors varied widely in their propensity to use the intended superordinate, a type of individual difference which is not readily explained within a theory where speakers are motivated in general to be efficient.

Our study suggests that robustness in communication is a product of both conventionality and compositionality working in conjunction (see Tamariz & Kirby, 2016). The benefits of linguistic conventions are manifest. Speakers’ use of compositional descriptions, on the other hand, offers its own advantage. First, using a flexible, combinatorial strategy may help speakers overcome bottlenecks in lexical access (see Levelt et al., 1999; Marslen-Wilson, 2001; Roelofs, 1992). Being able to convey categories through multiple strategies means that communication can proceed even if accessing particular lexical items (e.g., *appetizers*, *precipitation*) is fragile. Second, use of non-conventional strategies may be the result of Directors’ flexible construal of the categories articulated by the targets. The examples in Table 4 show that Directors were attuned to many different properties of the targets, some of which had similar meanings to *appetizers* (e.g., *common snacks you can share*), some of which did not (e.g., *salty foods without a bun*). The human ability to calculate what two items have in common is hugely flexible (Liu & Lupyan, 2023). It may be that suppressing these multiple construals and considering only those categories that are lexicalized in a single word involves additional communicative effort by Directors. In everyday life, context will influence which properties of the targets are most relevant (e.g., common ingredients vs. common food rituals). In the context of our task, Directors needed to decide for themselves which properties to highlight. Our study suggests that language users reduce communicative effort not only by reducing the length of their messages but also by favoring those construals of the world which are most conceptually prominent.

## CONCLUSION

People often wonder whether some ideas are better expressed in some languages than others. Although it is commonly asserted that all languages are mutually intertranslatable, such intertranslatability cannot be assumed but must be tested. Our study provides one such test: we find that communication is more successful when speakers can use a conventional term to convey a superordinate category. This suggests a practical limitation on what speakers convey when there is no linguistic convention to help them align their ideas. These results suggest that communication is robust because speakers can flexibly draw on both conventions and compositional resources when conveying meanings.

## ACKNOWLEDGMENTS

Thank you to Kevin Mui for JavaScript help and to Jing Paul, Kesong Cao, and Xiaoya Gong for help with online participant recruitment. We appreciate Xiaoya Gong for her hard work coding Chinese descriptions. Thank you to attendees of Experiments in Linguistic Meaning 2 and to members of the Lupyan Lab for their feedback on this work. Thank you to all study participants.

## AUTHOR CONTRIBUTIONS

Lilia Rissman: Conceptualization; Formal analysis; Investigation; Methodology; Project administration; Visualization; Writing—Original draft; Writing—Review & editing. Qiawen Liu: Conceptualization; Investigation; Methodology; Project administration; Visualization; Writing—Original draft. Gary Lupyan: Conceptualization; Formal analysis; Funding acquisition; Methodology; Project administration; Supervision; Visualization; Writing—Original draft; Writing—Review & editing.

## DATA AVAILABILITY STATEMENT

Stimuli materials, data files, analysis scripts, model syntax, and Supplementary Materials are available at: https://osf.io/cdg4j/.

## FUNDING INFORMATION

This research was supported by NSF PAC 2020969 awarded to G. Lupyan.

## Notes

^1^ There are different types of superordinate nouns, e.g., some are mass nouns (*wildlife*) and others count nouns (*animals*) (Wisniewski et al., 1996). Whether a particular category label has mass or count syntax also differs across languages (Takatori & Schwanenflugel, 1992). We do not, therefore, assume that superordinates constitute a homogenous lexical class. Indeed, the individual superordinates tested in this study differed in terms of the descriptions they elicited and how accurately they were communicated, a point we return to in Which Categories Were Easiest and Hardest to Convey? section.^2^ English data was collected before Chinese data, and we decided after English data collection to pay Chinese Directors and Matchers the same amount.^3^ Note that this regression estimate, derived from a mixed model with random intercepts and slopes, indicates that English pairs are less similar than Chinese pairs, an effect in the opposite direction from what is indicated by the raw means.^4^ This model excluded Participant by Term Language random slopes due to convergence failure.
